# Distinct neuronal populations in the basal forebrain encode motivational salience and movement

**DOI:** 10.3389/fnbeh.2014.00421

**Published:** 2014-12-04

**Authors:** Irene Avila, Shih-Chieh Lin

**Affiliations:** Neural Circuits and Cognition Unit, Laboratory of Behavioral Neuroscience, National Institute on Aging, National Institutes of HealthBaltimore, MD, USA

**Keywords:** basal forebrain, ventral pallidum, motivational salience, movement, fixation, rat

## Abstract

Basal forebrain (BF) is one of the largest cortically-projecting neuromodulatory systems in the mammalian brain, and plays a key role in attention, arousal, learning and memory. The cortically projecting BF neurons, comprised of mainly magnocellular cholinergic and GABAergic neurons, are widely distributed across several brain regions that spatially overlap with the ventral striatopallidal system at the ventral pallidum (VP). As a first step toward untangling the respective functions of spatially overlapping BF and VP systems, the goal of this study was to comprehensively characterize the behavioral correlates and physiological properties of heterogeneous neuronal populations in the BF region. We found that, while rats performed a reward-biased simple reaction time task, distinct neuronal populations encode either motivational salience or movement information. The motivational salience of attended stimuli is encoded by phasic bursting activity of a large population of slow-firing neurons that have large, broad, and complex action potential waveforms. In contrast, two other separate groups of neurons encode movement-related information, and respectively increase and decrease firing rates while rats maintained fixation. These two groups of neurons mostly have higher firing rates and small, narrow action potential waveforms. These results support the conclusion that multiple neurophysiologically distinct neuronal populations in the BF region operate independently of each other as parallel functional circuits. These observations also caution against interpreting neuronal activity in this region as a homogeneous population reflecting the function of either BF or VP alone. We suggest that salience- and movement-related neuronal populations likely correspond to BF corticopetal neurons and VP neurons, respectively.

## Introduction

Basal forebrain (BF) is one of the largest cortically-projecting neuromodulatory systems in the mammalian brain (Semba, 2000; Zaborszky, 2002; Jones, 2003), comprised not only of cholinergic neurons, but also of equally prominent GABAergic neurons and a smaller subset of glutamatergic neurons (Gritti et al., 1997, 2003; Henny and Jones, 2008). Through its modulation of cortical activity and plasticity, BF plays a key role in attention, arousal, learning and memory, and has been implicated in dementia and age-related cognitive decline (Everitt and Robbins, 1997; Wenk, 1997).

An important yet little recognized challenge facing the study of the BF is the anatomical overlap of multiple macrosystems. BF corticopetal neurons are widely distributed across several brain regions which overlap substantially with parts of the ventral striatopallidal system, including the ventral part of globus pallidus (GP) and the caudal part of VP (Heimer et al., 1991; Gritti et al., 1997, 2003). Given that the BF and ventral striatopallidal systems contain different cell types and distinct input-output connectivity patterns, the substantial overlap of two macrosystems raises the question of whether different neuronal groups in this region work together to achieve common functional goals, or whether they represent parallel neural systems that occupy the same anatomical space.

The spatial overlap of the two macrosystems also poses a challenge in interpreting experimental findings. For example, recent studies have identified a group of neurons in this region that encode the motivational salience of attended stimuli with robust bursting responses (Richardson and DeLong, 1991; Lin and Nicolelis, 2008). This motivational salience signal is correlated with faster and more precise decision speed (Avila and Lin, 2014), and is coupled with a frontal cortex event-related potential response with a short 5–10 ms delay (Nguyen and Lin, 2014). These results have been interpreted as reflecting the function of non-cholinergic BF corticopetal projections that amplify cortical activity associated with motivationally salient stimuli. However, other studies (Tindell et al., 2005, 2009; Smith et al., 2011) have interpreted similar neuronal activity as reflecting the output of VP neurons as they relay ventral striatum inputs to signal incentive salience of the cue, as well as modulating the impact of reward. These differing interpretations of the same neuronal activity highlight the lack of consensus on whether these neurons belong to the BF or VP system.

As a first step toward untangling the complexity of BF, the goal of this study was to comprehensively characterize the response profiles and physiological properties of heterogeneous neuronal populations in the BF region. Toward this end, we sought to identify the most common types of BF responses to key behavioral events, and to determine whether motivational salience and movement-related information are encoded by the same or different neuronal populations. We further investigated whether heterogeneous types of BF neurons possess distinct neurophysiological properties, such as firing rates, firing patterns and action potential waveforms. Identification of the heterogeneous behavioral and neurophysiological phenotypes of BF neurons will provide the necessary information for future studies to determine the neurochemical identity and their associated macrosystems.

## Materials and methods

All experimental procedures were conducted in accordance with the National Institutes of Health (NIH) Guide for Care and Use of Laboratory Animals and approved by the National Institute on Aging Animal Care and Use Committee. Detailed experimental procedures have been described in full in our recently published report (Avila and Lin, 2014). The current paper represents additional analysis of the neurophysiology data collected from that study.

### Subjects

Six male Long Evans rats (Charles River, NC), aged 3–6 months and weighed 300–400 grams at the start of the experiment, were used in this study. These rats were first trained in the reward-biased simple RT task and subsequently underwent surgery for chronic neuronal activity recording. Rats were housed under 12/12 h day/night cycle with *ad libitum* access to rodent chow and water in environmentally controlled conditions. During training and recording procedures, rats were mildly water restricted to 90% body weight, and were trained in a daily session of 60–90 min in length, five days a week. Rats received 15 min water access at the end of each training day with free access on weekends.

### Reward-biased simple RT task

Behavioral training and neural activity recording were conducted in operant chambers (Med Associates Inc., VT), equipped with one photo-beam lickometer reward port (CT-ENV-251L-P) located in the center of the front panel, flanked by two nosepoke ports (ENV-114M). Only the right nosepoke port was used in behavioral training as the fixation port. Each chamber was equipped with two ceiling-mounted speakers (ENV-224BM) to deliver auditory stimuli, and two stimulus lights (ENV-221) positioned above the reward port in the front panel. Behavior training protocols were controlled by Med-PC software (Version IV).

**Figure 1:**
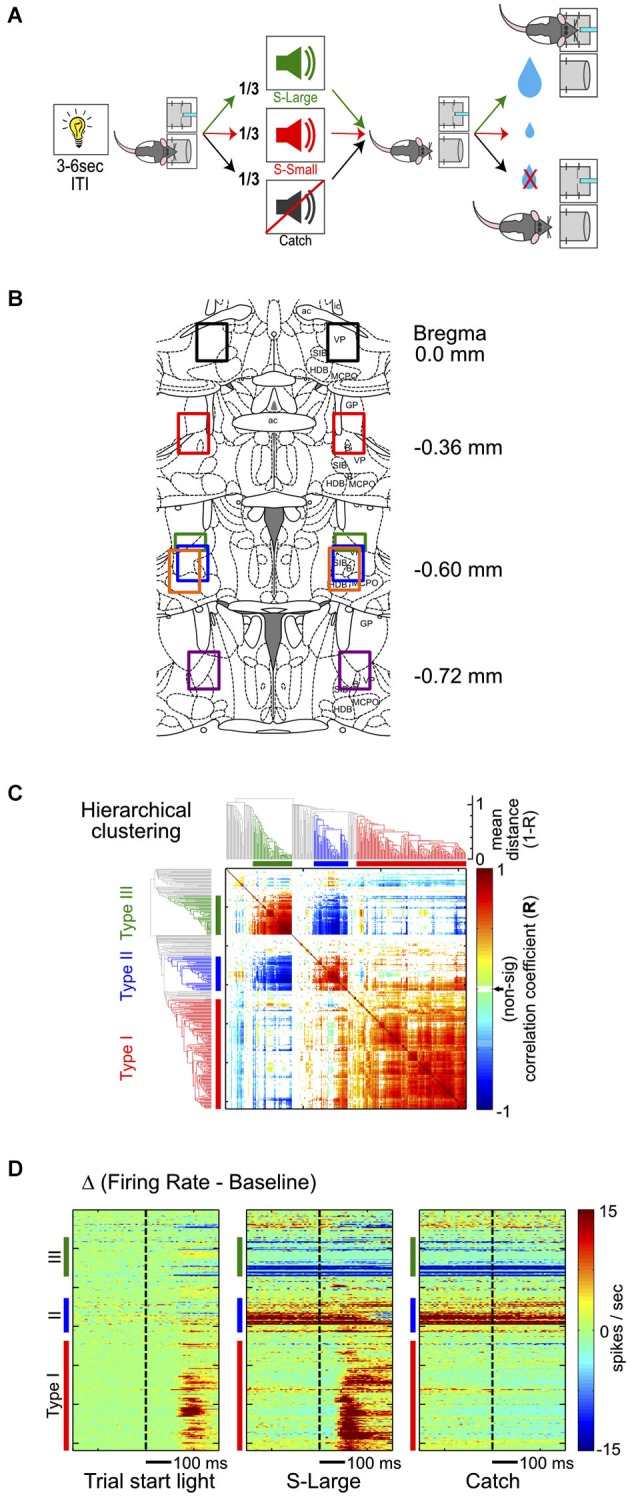
**Hierarchical clustering analysis of BF neuronal response types. (A)** Schematic of the reward-biased simple RT task. Each trial is initiated by a house light signal, prompting rats to enter a nosepoke fixation port. Rats encounter three possibilities while maintaining fixation: (1) S-Large sound predicting 4 drops of water in the adjacent reward port; (2) S-Small sound predicting 1 drop of water; or (3) Catch trials with no sound and no reward. **(B)** Histological reconstruction of electrode locations. Each color represents the reconstructed recording area from one rat. **(C)** Hierarchical clustering analysis of all BF neurons based on the similarity of neuronal responses and the associated dendrogram. The clustering algorithm identifies three major types of responses in BF neurons, identified by the same colors in all figure panels: Type-I, red; Type-II, blue; Type-III, green. **(D)** Three response features from each neuron are used in the hierarchical clustering algorithm, sorted based on neuronal order in the dendrogram. The three features are PSTHs in the [−300, 300]ms window relative to the trial-start light signal, S-Large sound and the Catch stimulus, with baseline firing rates subtracted.

In the reward-biased simple RT task, rats were shaped to nosepoke in the fixation port and maintain fixation until tone presentation. Four different foreperiods (0.35, 0.5, 0.65 and 0.8 s) were used and were pseudo-randomly varied across trials to minimize temporal expectation of stimulus onset. While maintaining fixation in the fixation port, rats were presented with three different conditions with equal probability: (1) white noise; (2) 100 Hz clicker sound; or (3) catch trials (Figure 1A). The two auditory stimuli were chosen to be clearly discriminable and presented at a suprathreshold level (2 s, 80 dB) to minimize sensory detection and discrimination effort. The two sounds were associated with either 1 vs. 4 or 4 vs. 1 drops of water in a session, and no reward was delivered in catch trials. Since the sound associated with large reward was counter-balanced across animals, the sound predicting the larger reward will be referred to as S-Large, while the sound predicting the smaller reward referred to as S-Small.

Water reward was delivered starting at the 3rd lick. Only trials with the 3rd lick completed within a 3 s reward window were defined as successful Go response and rewarded. Early fixation port exit before the foreperiod resulted in no reward delivery. The inclusion of catch trials ensured that tone onset was the most reliable predictor of reward and that rats did not employ a timing strategy for responding.

The inter-trial interval (ITI) was 3–6 s, signaled by a white stimulus light. The offset of the light thus served as the trial start signal, indicating that fixation port entry could now lead to tone presentation. Premature fixation port entry and premature licking both resulted in resetting the ITI timer.

### Stereotaxic surgery and electrode

After reaching asymptotic behavioral performance, rats were taken off water restriction for at least 3 days before undergoing stereotaxic surgery for chronic electrode implant. Rats were anesthetized with isoflurane (2–4% isoflurane induction followed with 1–2% maintenance) and received atropine (0.02–0.05 mg/kg, i.m.) to reduce respiratory secretion.

Custom-built 32-wire multi-electrode moveable bundles were implanted bilaterally into BF. The electrodes consisted of two moveable bundles, each containing 16 polyimide-insulated tungsten wires (California Fine Wire, CA) controlled by a precision microdrive. Eight of the wires in a bundle were 38 μm in diameter and the other 8 were 16 μm diameter, with 0.1–0.3 MΩ impedance measured at 1 kHz (niPOD, NeuroNexusTech, MI). The two cannulae of the electrode were precisely positioned to target the BF on both hemispheres at AP −0.6 mm, ML ± 2.25 mm relative to Bregma (Paxinos and Watson, 2007). During surgery, the cannulae were lowered to DV 6–6.3 mm below cortical surface using a micropositioner (Model 2662, David Kopf Instrument), and the electrodes were advanced to 7 mm below cortical surface. After reaching target depth, the electrode and screws were covered with dental cement (Hygenic Denture Resin). One skull screw over the cerebellum served as the common electrical reference, and a separate screw over the opposite cerebellum hemisphere served as the ground.

Rats received acetaminophen (300 mg/kg, oral) and topical antibiotics after surgery for pain relief and prevention of infection. Water restriction and behavioral training resumed 7–10 days after surgery. Cannulae and electrode tip locations were verified with cresyl violet staining of histological sections at the end of the experiment and compared with reference anatomical planes (Paxinos and Watson, 2007). All electrodes were found at expected positions (Figure 1B).

### Recording

Recording sessions were conducted with electrodes located across multiple subregions, including the ventral part of GP, posterior part of VP, substantia innominata (SI), nucleus basalis of Meynert (NBM, or B), magnocellular preoptic nucleus (MCPO), and horizontal limb of the diagonal band (HDB), consistent with the spatial distribution of cortically projecting BF neurons (Gritti et al., 1997).

Each recording session lasted 60–90 min. At the conclusion of each recording session, BF electrodes were advanced at least 100 μm and 3–7 days elapsed before the next recording session. One recording session at each electrode depth was included in the final analysis and therefore sampled distinct BF single neuron ensembles. A total of 309 BF single units were recorded from 40 sessions in 6 rats, at DV 7.1–8.3 mm below cortical surface (Figure 1B).

Electrical signals were referenced to a common skull screw placed over the cerebellum. Electrical signals were filtered (0.3 Hz–7.5 kHz) and amplified using Brighton Omnetics or Cereplex M digital headstages and recorded using a Neural Signal Processor (Blackrock Microsystems, UT). Single unit activity was further filtered (250 Hz–5 kHz) and recorded at 30 kHz. Spike waveforms were sorted offline using Offline Sorter (Plexon Inc., TX). Only single units with clear separation from the noise cluster and with minimal (<0.1%) spike collisions (spikes with less than 1.5 ms inter-spike interval) were used for further analyses. Additional cross correlation analysis was used to remove duplicate units recorded simultaneously over multiple electrodes.

### Analysis

**Figure 2:**
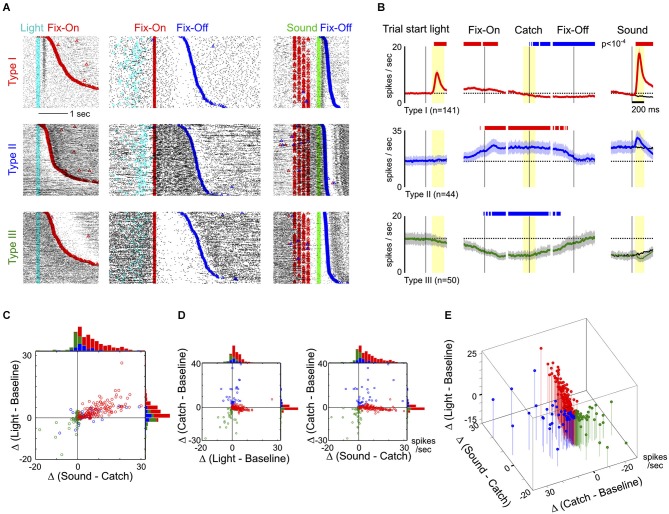
**Response features of the three main types of neurons in the BF region. (A)** Raster plots of activity from three example neurons, one in each row, from the three types of BF neurons. Individual trials were aligned to trial-start light signal and sorted by the latency to enter fixation port (Fix-On; left column), or aligned to Fix-On in Catch trials and sorted by the latency to exit fixation port (Fix-Off; middle column), or aligned to sound onset and sorted by reaction time (right column). **(B)** Average PSTHs (± s.e.m.) for the three types of BF neurons in response to different trial events, indicated by the black vertical line. The left column shows responses to the trial-start light signal. The middle three columns show responses to three events in Catch trials: Fix-On, the Catch stimulus (Catch) and Fix-Off. The right column shows responses to sound stimuli, with responses to the Catch stimulus shown in black for comparison. Dashed lines depict the average baseline firing rates for each type of neuron. Significant changes in firing rates are indicated by red (increased rate) and blue (decreased rate) bars. In the left four columns, firing rates are relative to baseline firing rates; in the right column, they are relative to catch trials (paired *t*-tests, *p* < 10^−4^). **(C)** Scatter plot of individual neuronal response amplitudes to the trial start light (relative to baseline) vs. sounds (relative to catch stimulus), along with marginal distributions. The time windows for response amplitude calculation are indicated by yellow shaded areas in **(B)**. There was substantial overlap between Type-I and II neurons. **(D)** Scatter plots of individual neuronal response amplitudes between light vs. catch stimulus and sound vs. catch stimulus. Conventions are the same as in **(C)**. **(E)** The three features combined separates the three main types of BF neurons.

**Figure 3:**
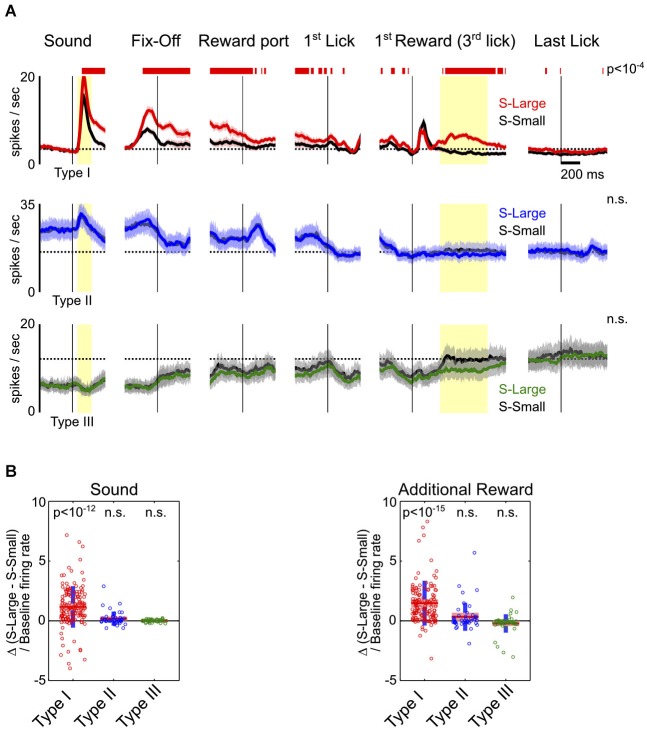
**Motivational salience information is specific to Type-I BF neurons. (A)** Average PSTHs for the three types of BF neurons in S-Large (colored) vs. S-Small (black) trials. Significant response modulations between the two trial types, reflecting the influence of motivational salience, were found only in Type-I BF neurons, and persisted throughout all behavioral epochs. Conventions are the same as in Figure 2B. **(B)** The relative difference of response amplitudes between the two trial types at sound onset and the 2nd–4th rewarded licks. Time windows for analysis indicated by yellow shaded areas in **(A)**. Type-I BF neurons showed stronger responses to S-Large and to additional reward (*t*-tests for significant differences from zero). Other neuron types did not show preference for either S-Large or S-Small stimuli.

**Figure 4:**
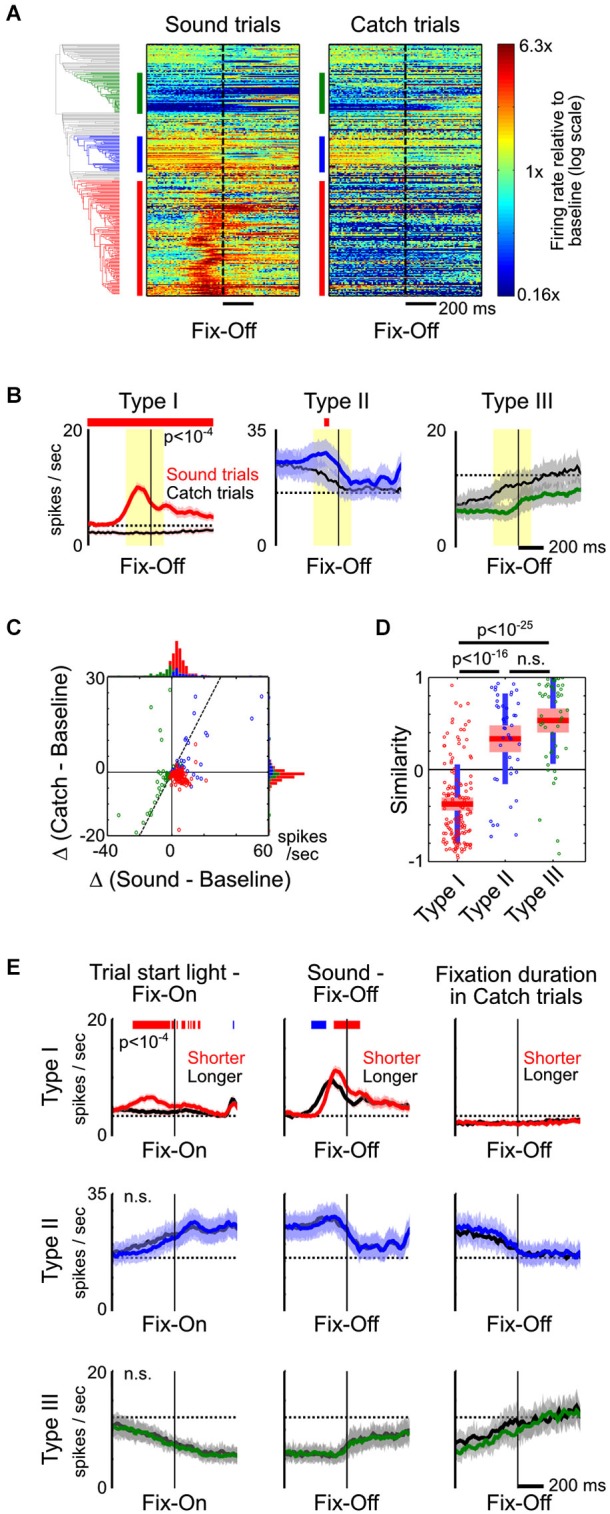
**Type-II and III BF neurons reflect fixation and movement states. (A)** PSTHs of all individual BF neurons to fixation port exit (Fix-Off) in sound trials and catch trials, with response amplitude normalized by their respective baseline firing rates. While the response of Type-I BF neurons were the opposite in sound and catch trials, the responses of other neurons were highly similar when animals exited the fixation port, irrespective of whether the exit response was prompted by a stimulus. **(B)** The average PSTHs of the three types of BF neurons relative to fixation port exit response, in sound trials (colored) and catch trials (black). Significant response modulations between the two trial types are indicated by the red line (paired *t*-tests, *p* < 10^−4^). **(C)** Scatter plot of the response amplitude of individual BF neurons in sound vs. catch trials, calculated at the [−200,100] ms window relative to fixation exit (yellow shaded area in **B**). Many Type-II and III neurons had similar response amplitudes in both trial types and fell along the unity line (dashed line), while Type-I neurons were in the opposite quadrants. **(D)** Similarity of fixation port exit response temporal profiles between the two trial types (ANOVA, *F*_(2,232)_ = 94.1, *p* < 10^−29^, *post hoc* *t*-tests). **(E)** The average PSTHs of the three types of BF neurons aligned to fixation port entry (left) or exit (middle and right), for trials with shorter (colored) or longer (black) response latencies. The respective response latencies are indicated above each column. Significant response differences between shorter and longer latency trials are indicated by the red/blue line (paired *t*-tests, *p* < 10^−4^).

Behavioral and neural data were analyzed using custom scripts in Matlab 2013a (Mathworks, MA) and statistical analyses were performed with SPSS (Version 20, IBM Corp). Statistical tests and associated *p*-values are indicated in the text and figure legends. Error bars represent standard error of the mean (s.e.m.) unless otherwise stated. Significant differences in average PSTHs between two trial types (Figures 2B, 3A, 4B) were determined by paired *t*-tests between each 10-ms bin, with Bonferroni’s correction for multiple comparisons (α/n; *α* = 0.01, *n* = 100, corrected *p* = 10^−4^).

### Hierarchical clustering analysis

The hierarchical clustering analysis groups BF neurons based on the similarity of their responses to three key events: (1) trial start light offset; (2) S-Large onset; and (3) the timestamp of would-be stimulus onset (i.e., catch stimulus) in catch trials. A peri-stimulus time histogram (PSTH) was generated for each BF neuron against each event at [−0.3, 0.3 ] s window with 10 ms bins, consisting a 60-element vector. The baseline activity, defined as the average firing rate at [−2,−1] s window relative to trial start light offset, was subtracted from each PSTH to reflect the modulation of firing rates relative to respective baselines. For each BF neuron, the three baseline-subtracted PSTHs were concatenated to form a 180-element vector. The similarity of responses between two neurons, ***r***, was defined as the cosine of the angle between the 180-element vectors, which was calculated using the coefficient option in the xcorr.m function in Matlab, and was bounded between [−1,1]. The pairwise similarity index was statistically significant if the value exceeded the [0.1%, 99.9%] confidence interval defined by 10,000 random shuffling of the 180-element vectors. Non-significant similarities measures were assigned as zeros to minimize the contribution of noise in the clustering analysis.

The clustering analysis was based on linkage.m function in Matlab, using unweighted average distance as the distance between clusters. The distance measure was defined as 1−***r***, which had a range of [0,2]. A shorter distance means that two neurons are more similar (with ***r*** closer to 1), while dissimilar neurons have longer distance measures. The resulting dendrogram was sorted based on the mean square ***r*** values associated with each neuron.

### Inter-spike interval (ISI) analysis

**Figure 5:**
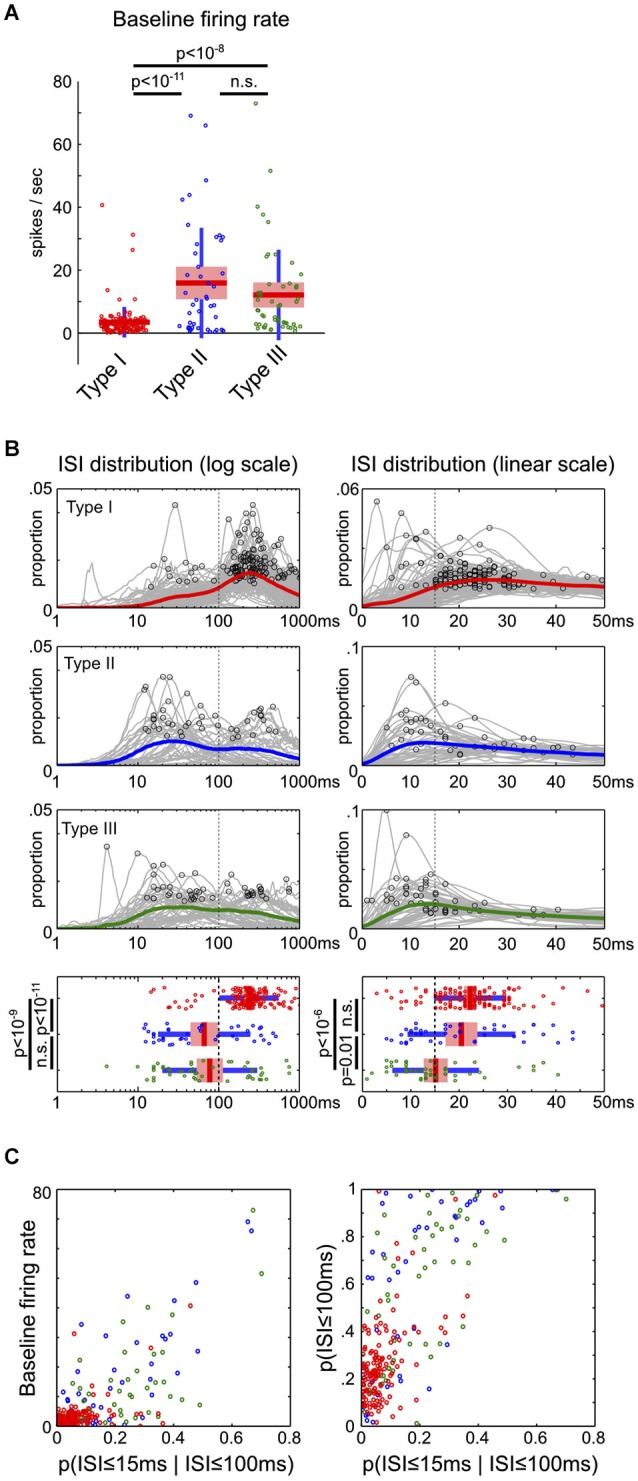
**Firing rate and inter-spike interval characteristics of BF neuronal types. (A)** The baseline firing rates of Type-II and III neurons were significantly higher than Type-I BF neurons (ANOVA, post hoc *t*-tests). **(B)** ISI distributions on both log and linear scales for the three types of BF neurons. The mean ISI distributions for each type of neurons are shown in colored lines, with the peak of individual ISI distributions indicated by black circles. Significant differences in the peak of ISI distributions were found between the three types of neurons (ANOVA, log scale, *F*_(2,232)_ = 35.27, *p* < 10^−13^; linear scale, *F*_(2,232)_ = 12.74, *p* < 10^−5^, post hoc *t*-tests). Type-I BF neurons showed two distinct peaks in the ISI distributions: 200–300 ms on the overall log scale ISI distribution, and 15–30 ms on the zoomed-in linear scale ISI distribution. Vertical dashed lines indicate the boundaries used to separate the three types of BF neurons. (**C)** Scatter plots between baseline firing rates and ISI distribution features. Type-I neurons are reliably discriminated from Type-II and III neurons based only on firing rate and ISI features. In addition to low baseline firing rates, Type-I BF neurons had lower proportions of short ISIs, generally with *p*(ISI ≤ 100 ms) less than 0.4, and *p*(ISI ≤ 15 ms | ISI ≤ 100 ms) less than 0.15.

Two ISI distributions in log and linear scales were generated for each neuron. The log-scale ISI distribution sorted ISIs between 1 ms to 2 s into 200 bins, and smoothed with a 3-element Hanning window. The global peak of each log-scale ISI distribution was identified and compared in Figure 5B. The linear-scale ISI distribution sorted ISIs between 0 ms to 100 ms into 100 bins, and smoothed with a 6-element Hanning window. The first local peak with at least 80% of the maximum magnitude in the [0,50] ms window was identified and compared in Figure 5B.

### Action potential waveform analysis

**Figure 6:**
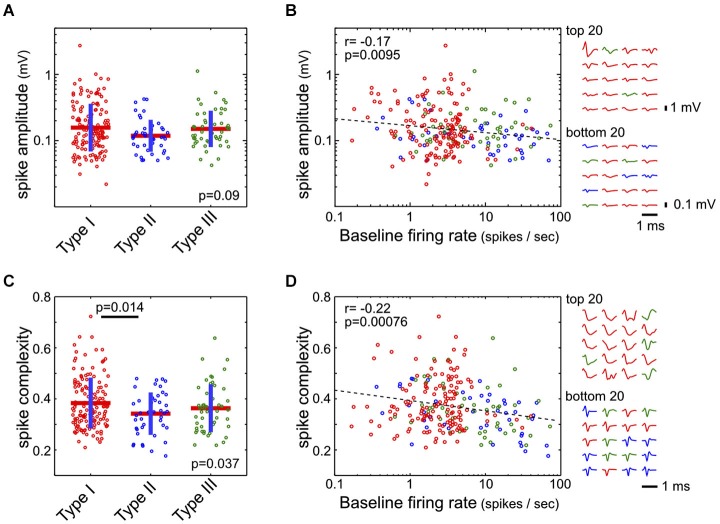
**Spike amplitude and complexity in the three types of BF neurons. (A)** Spike amplitudes were not statistically different between the three types of BF neurons (ANOVA, *F*_(2,232)_ = 2.43, *p* = 0.09). **(B)** However, spike amplitude was negatively correlated with the baseline firing rate (Pearson correlation, *r* = −0.17). Spike waveforms for the BF neurons with highest and lowest amplitudes are shown on the right, with different amplitude scale bars. **(C)** Spike complexity, defined as the mean absolute amplitude of the peak normalized waveform, was higher in Type-I than in Type-II neurons (ANOVA, *F*_(2,232)_ = 3.36, *p* = 0.037; significant post hoc *t*-test indicated). **(D)** Spike complexity showed a stronger dependency with baseline firing rate (Pearson correlation, *r* = −0.22). Normalized spike waveforms for the 20 BF neurons with the most and least complex spike waveforms are shown on the right.

Waveform features—both amplitude and complexity—were extracted from the average waveform of each single unit. The spike amplitude was defined as the difference between the maximum and minimum of the waveform. The spike complexity measure was designed as a proxy for estimating the width of the action potential waveform, which were often complex and exceeding the 1 ms action potential recording window. The action potential waveform ***w*** was first normalized by its peak value max(abs(***w***)), and spike complexity was defined as the mean absolute value of this normalized waveform. In other words, the spike complexity of a waveform ***w*** is defined as mean(abs(*w*/max(abs(***w***)))). Under this definition, short spikes with waveforms quickly returning to baseline should have lower spike complexity, while longer and complex spikes should have higher spike complexity (Figure 6D).

## Results

To study the behavioral correlates of BF neuronal responses in a well-controlled context, we used the reward-biased simple RT task (Figure 1A) described in a recent study by Avila and Lin (Avila and Lin, 2014). In this task, each trial started with a light signal that instructed rats to enter a nosepoke fixation port. While maintaining fixation, rats encountered three equally likely scenarios: a sound (S-Large) predicting four drops of water reward, a different sound (S-Small) predicting one drop of water reward, or no sound (Catch) and no reward. After sound onset, rats exited the fixation port quickly and moved to collect reward in the adjacent reward port. Detailed behavioral response characteristics in this task have been previously described (Avila and Lin, 2014). We found rats responded faster in S-Large trials than in S-Small trials, indicating that the stimulus paired with a larger reward was motivationally more salient (Avila and Lin, 2014). This task provided a rich and well-controlled behavioral context to investigate whether the activity of BF neurons are modulated by several important behavioral variables, including the motivational salience of the stimulus, the motor response, as well as the state of maintaining fixation.

Six male Long-Evans rats were trained in the reward-biased simple reaction time (RT) task and 309 well-isolated single units were recorded in the BF region over 40 sessions (Avila and Lin, 2014). BF neurons were recorded throughout brain regions containing corticopetal BF neurons (Gritti et al., 1997), including SI, NBM, MCPO, and HDB, as well as the ventral part of GP and the caudal part of ventral pallidum (VP) which are part of the ventral striatopallidal circuit (Figure 1B).

To characterize the response profile of all recorded neurons in this region, we first focused on their responses to three major behavioral events: (1) the trial-start light signal; (2) reward-predicting sounds; and (3) nosepoke fixation in the absence of sound (catch trials). To identify the most common response profiles and categorize BF neurons into distinct groups without *a priori* defining the relevant features in their response profiles, we chose the hierarchical clustering analysis to explore the similarity of neuronal responses (Figure 1C). The hierarchical clustering analysis converts the similarity of neuronal responses between all BF neuronal pairs into an easily accessible tree structure in which neurons with similar response profiles are clustered together. This analysis identified three main types of BF neurons, collectively accounting for 76% of all recorded neurons (141, 50 and 44 of 309 total neurons; Figure 1C). The response patterns of individual BF neurons were highly similar within each cluster and distinct between clusters (Figure 1D). Subsequent analyses focused on the three most common BF response types to avoid ambiguity associated with additional smaller clusters of neurons.

To understand the response features that defined the three main types of BF neurons, we next examined their responses relative to the three key events (Figures 2A,B). Three example neurons from each type are illustrated in Figure 2A. The largest group of BF neurons (46%, 141/309), referred to as Type-I neurons, showed robust bursting responses to both the trial-start light and reward-predicting sounds. The overall proportion of this group of neurons, as well as their response patterns, are similar to the salience-encoding BF neurons that we have previously described (Avila and Lin, 2014). In contrast, Type-II (14%, 44/309) and Type-III (16%, 50/309) neurons showed no response to either light or sounds, but showed sustained firing increases or decreases, respectively, during nosepoke fixation (Figure 2B).

While Type-I neurons were characterized by distinct phasic excitatory responses to light and sounds, the presence of phasic excitatory responses alone was not sufficient to fully differentiate Type-I from Type-II neurons, as some Type-II neurons also showed low amplitude excitatory responses to both stimuli (Figure 2C). To completely distinguish the three types of neurons, it was necessary to include the neuronal responses during nosepoke fixation in our analysis (Figures 2D,E). These results show that the hierarchical clustering analysis is not only able to identify the most prevalent salience-encoding neurons in the BF, but also uncovers two additional types of BF neuronal response that can be distinguished from salience-encoding BF neurons based on their responses during fixation.

To further investigate how BF neuronal responses are modulated by motivational salience signal, we compared BF neuronal responses between S-Large and S-Small trials. These trials shared highly similar motor responses and differed only in the associated motivational salience. We found that motivational salience significantly modulated response amplitudes of Type-I, but not Type-II or Type-III, BF neurons (Figure 3A). While Type-II and III BF neurons also showed dynamic modulations related to key behavioral events, such as fixation port exit, reward port entry and the onset of licking, such responses were not modulated by motivational salience (Figures 3A,B). The modulation of Type-I neuronal responses by motivational salience persisted throughout the entire response epoch (Figure 3A). Furthermore, Type-I neurons were the only neuronal group with phasic bursting responses to the first drop of reward delivery. Type I neurons also showed excitatory responses to the 2nd–4th drops of water reward in S-Large trials (Figure 3A). The large variability of response amplitude between S-Large and S-Small trials in Type-I neurons (Figure 3B) likely reflects the variability of associated motivational salience in individual sessions, which is coupled with the initial decision time to make the rapid fixation port exit response (Avila and Lin, 2014). These observations support the conclusion that Type-I neurons represent a distinct population in the BF whose activity is modulated by motivational salience throughout all behavioral epochs.

On the other hand, the response profiles of Type-II and III neurons suggest that they are not modulated by motivational salience, but instead are likely modulated by movement and fixation state. For example, in catch trials, firing rates of Type-II and III neurons increased and decreased, respectively, while rats maintained fixation in the nosepoke port (Figures 2A,B). To further test whether the activity of Type-II and III neurons is primarily modulated by movement and fixation state, we directly compared neuronal responses around fixation port exit in the presence or absence of a preceding sound stimulus. This allowed us to investigate how BF neurons respond to the same change in movement and fixation state in two very different scenarios. We found that, while Type-I neurons showed the opposite pattern of responses relative to baseline firing rate around the time of fixation port exit during sound trials and catch trials, other BF neurons showed largely similar response profiles in the two trial types (Figures 4A,B). Both the response amplitudes and their temporal patterns around fixation port exit were highly similar in Type-II and III neurons, but dissimilar in Type-I BF neurons (Figures 4C,D). These results support that Type-II and III neurons modulate their activity based on movement and fixation state, regardless of whether the fixation port exit behavior was prompted by a motivationally salient stimulus.

Despite the highly similar response patterns of Type-II and III neurons around fixation port exit in Sound and Catch trials, we sought to further understand whether the numerical differences in their response amplitudes may reflect differences in associated movement speed. We therefore compared neuronal activity time-locked to fixation port entry or exit between trials with similar motivational salience levels but with different response latencies (Figure 4E). While there remained differences in movement speed between trials in each of the three comparisons, the differences in movement speed were much smaller than the difference between Sound and Catch trials analyzed in Figures 4A–D. It is expected that movement-related neuronal activity should show little or no modulation in these comparisons. Indeed, we found that in all three cases, Type-II and Type-III neurons showed highly similar response patterns time-locked to movement onsets. The tight coupling with movement and fixation onsets supports the idea that Type-II and III neurons are directly related to movement and fixation. On the other hand, the activity of Type-I neurons was differentially modulated at movement onsets, similar to our observations in Figures 3A, 4B. This suggests the motivational salience information carried by Type-I neuron likely plays a role in modulating the speed of decision making but does not directly control the precise timing of movement onset (Avila and Lin, 2014).

If the three types of BF neurons represent distinct groups of neurons in the circuit, they may also have different intrinsic neurophysiological characteristics. To test this prediction, we characterized the baseline firing rates and inter-spike interval (ISI) distributions of the three types of BF neurons. The baseline firing rates were significantly different between neuronal types (ANOVA, *F*_(2,232)_ = 28.2, *p* < 10^−10^): while most Type-I neurons were physiologically homogeneous and had baseline firing rates less than 10 spikes/s (135/141), many Type-II and III neurons had high baseline firing rates >10 spikes/s (Type-II, 22/44; Type-III, 21/50; Figure 5A). In other words, high firing neurons were more likely Type-II or III neurons, while low firing neurons were more likely Type-I salience-encoding BF neurons. Furthermore, low firing Type-I BF neurons showed two distinct peaks in their ISI distributions: one at 200–300 ms corresponding to their average baseline firing rates, and another peak at 15–30 ms corresponding to their intra-burst dynamics (Figure 5B). Type-II and III neurons had significantly shorter ISI peaks than Type-I neurons (Figure 5B). While there was a large variability in both the baseline firing rates and ISI distributions of Type-II and III neurons, they can be largely separated from Type-I neurons (Figure 5C). These observations support that the three types of BF neurons have different neurophysiological characteristics. In particular, Type-I BF neurons can be largely distinguished from other groups based on neurophysiological properties alone.

Finally, we asked whether there were distinct action potential waveform features associated with the three types of BF neurons. While there was no significant difference between the three types of neurons in their spike amplitude (ANOVA, *F*_(2,232)_ = 2.43, *p* = 0.09, Figure 6A), there was a subtle, although significant, relationship (*r* = 0.-17) between spike amplitude and baseline firing rate, such that low firing neurons tended to have larger spikes (Figure 6B). We noted that many neurons had long spike waveforms that could not be fully captured by the 1 ms spike window, as well as complex waveform shapes that made estimation of spike features difficult. As a proxy for estimating the duration of action potentials, we used the spike complexity measure, which was defined as the mean absolute amplitude of the peak-normalized spike waveform. Under this definition, narrow spikes have lower complexity while broader spikes have higher complexity. We found that Type-I neurons had a slightly higher spike complexity, compared to Type-II neurons (ANOVA, *F*_(2,232)_ = 3.36, *p* = 0.037; post hoc *t*-test *p* = 0.014; Figure 6C). Furthermore, there was a significant dependency of spike complexity on baseline firing rate (*r* = −0.22; Figure 6D), such that low firing BF neurons tended to have broader and more complex spikes. These results support that low-firing salience-encoding BF neurons (Type-I) were commonly associated with large, broad, and complex spike waveforms.

## Discussion

This study aims to provide the first comprehensive characterization of behavioral correlates and neurophysiological properties for neurons in the BF region. Our results establish that the response profiles of BF neurons are heterogeneous, as we identify three major types of neuronal responses in the BF (Figure 1). We found that motivational salience information is carried selectively by one specific type of neuron, Type I (Figures 2, 3), whose characteristics are consistent with the salience-encoding BF neurons recently reported (Lin and Nicolelis, 2008; Avila and Lin, 2014; Nguyen and Lin, 2014). In addition, we identified two other types of neurons, Type II and III, whose activity is not modulated by motivational salience, but rather is mainly modulated by fixation and movement state (Figures 2, 3, 4). The three types of neurons differ in intrinsic neurophysiological properties, including firing rates and ISI distributions (Figure 5). Furthermore, slow firing salience-encoding BF neurons have, on average, larger, broader and more complex spike waveforms (Figure 6). Together, these findings support the conclusion that multiple physiologically distinct groups of neurons coexist in the BF region that are functionally independent of each other.

Our findings that describe the physiological and functional heterogeneity of BF neurons are important as they support the underlying anatomical heterogeneity in this region. BF is anatomically complex on at least two different levels. First, BF contains multiple populations of magnocellular corticopetal projection neurons, including cholinergic, GABAergic and glutamatergic neurons (Gritti et al., 1997, 2003). Second, these diverse groups of projection neurons spatially overlap with parts of the ventral striatopallidal regions, including the ventral part of GP and the caudal portion of VP (Heimer et al., 1991; Gritti et al., 1997, 2003). While the first level of complexity has been well recognized in the BF literature (Sarter and Bruno, 2002; Lin et al., 2006; Lau and Salzman, 2008; Lin and Nicolelis, 2008), the second level of complexity—the spatial overlap between the BF corticopetal system and the ventral striatopallidal system—has received little recognition in the neuroscience literature. The anatomical overlap of these two macrosystems presents challenges when interpreting recording, lesion, and pharmacological results from either the BF or VP, as manipulations of this region likely influence multiple neuronal populations within both macrosystems. Furthermore, neurophysiological activity in this brain region may have been interpreted differently in the literature according to different assumptions that observed neuronal activity represents either BF or VP activity (more discussion later). The current study cautions against treating all neurons in the BF region as a homogeneous population (Tindell et al., 2005, 2009; Smith et al., 2011; Thomson et al., 2014; Tingley et al., 2014), which likely conflates the respective contributions of BF and VP macrosystems.

To classify BF neuronal response profiles, we used the hierarchical clustering analysis. The hierarchical clustering analysis is only one of many methods for classification, and has both strengths and limitations. The strength of this method is its ability to uncover regularities in a high-dimensional dataset and, in this specific case, reveal that neuronal responses during nosepoke fixation provide a key new dimension that distinguishes Type-II and III neurons from Type-I BF neurons. The limitation of this method in the current application is that the exact cluster membership for a specific neuron may vary depending on both the choice of behavioral epochs for PSTHs and the choice of the similarity measure. Despite the ambiguity regarding the exact cluster affiliation for a given neuron, the core of the three types of BF neurons we describe here remains commonly clustered together irrespective of the choice of parameters. Our analysis therefore has focused on the three most common and robust types of BF responses to avoid ambiguity associated with additional smaller clusters of neurons. Overall, we view the hierarchical clustering analysis as an important tool that provides a heuristic starting point to explore and uncover stereotypical response patterns in our dataset. The validity of distinctly classifying three types of BF neurons is supported by their similar responses to other behavioral events not used in the clustering analysis (Figures 3, 4), as well as their broadly segregated physiological characteristics including baseline firing rate, the fine temporal structure of spike trains and their waveforms (Figures 5, 6). These results support that the three types of neurons likely correspond to distinct populations of neurons in the BF region, and provide the neurophysiogical basis for future neurochemical identification of the different cell types.

The most prominent Type-I BF neuron matches the salience-encoding neurons recently described (Lin and Nicolelis, 2008). These neurons are considered non-cholinergic BF neurons because their average firing rates are not modulated between waking and sleep states (Lin et al., 2006; Lin and Nicolelis, 2008), unlike the cholinergic BF neural population (Lee et al., 2005). Their bursting responses to stimuli reflects the strength of motivational salience, and is coupled with faster and more precise decision speed (Avila and Lin, 2014). Furthermore, bursting activity generates a frontal cortex ERP response with a delay of 5–10 ms (Nguyen and Lin, 2014), consistent with BF corticopetal projecting neurons with conduction latencies less than 5 ms (Aston-Jones et al., 1985; Reiner et al., 1987). The current study extends these previous findings and shows that Type-I BF neurons represent the only group of BF neurons that carry information about motivational salience. Moreover, motivational salience information is reflected not only during the initial phasic bursting response, but subsequently influences neuronal activity throughout all response epochs. We also found that the slow-firing salience-encoding neurons have large action potential amplitudes, as well as broader and more complex spike waveforms, consistent with these neurons being the large magnocellular projecting neurons in this area (Gritti et al., 1993, 1997). Given that we have been able to record functionally and physiologically highly homogeneous neurons not only in ventral GP and caudal VP, but more broadly throughout SI, NBM and MCPO, our results suggest that Type-I salience-encoding neurons likely correspond to a distinct population of corticopetal BF neurons, rather than VP neurons. The non-cholinergic identity of these cells led us to hypothesize that Type-I salience-encoding BF neurons are most likely the large parvalbumin positive GABAergic corticopetal neurons that have been previously identified in the BF region (Gritti et al., 1997, 2003; Lin et al., 2006; Henny and Jones, 2008; Lin and Nicolelis, 2008; Avila and Lin, 2014; Nguyen and Lin, 2014).

Several recent studies (Tindell et al., 2005, 2009; Smith et al., 2011) have recorded from the same stereotaxic coordinates in rats as we have, targeting the caudal part of VP that corresponds to a hedonic hotspot identified in pharmacological studies (Smith and Berridge, 2005; Peciña et al., 2006). While those studies did not classify neurons into distinct groups, the main neuronal responses reported in those studies are highly similar to the salience-encoding neurons that we describe in the current study, with low baseline firing rates and phasic bursting responses both to reward-predicting cues and to reward delivery. We therefore suggest that the results from those studies likely reflect the contributions of salience-encoding BF neurons and should be interpreted in the context of the BF corticopetal projection system, rather than reflecting the functions of the ventral striatopallidal system.

Type-II and III neurons, on the other hand, are not modulated by motivational salience. Instead, their activity is directly coupled with fixation and movement states, and respectively increase and decrease firing rates while rats maintained fixation. These two groups of neurons mostly have higher firing rates and small, narrow action potential waveforms. These features are similar to the properties of GP neurons described in both monkeys and rodents (DeLong, 1971; Gardiner and Kitai, 1992; Kelland et al., 1995; Shi et al., 2004; Jin et al., 2014). First, GP neurons commonly have high baseline firing rates. Second, the activity of GP neurons is coupled with movement. Third, movement-related modulation of GP activity can be excitatory or inhibitory, and such modulation is commonly sustained during an action sequence (Jin et al., 2014). The opposite responses associated with the same movement events in Type-II and III neurons are also similar to the “action-on” and “action-off” neurons in another pallidal structure, substantia nigra pars reticulate (SNr; Fan et al., 2012). Lastly, similar movement-related activity modulations have been observed in studies targeting the VP (Root et al., 2013). Taken together, these observations suggest that Type-II and III neurons are physiologically and functionally similar to other neurons in the GP and VP. Based on these observations, we hypothesize that Type-II and III neurons likely belong to the ventral striatopallidal system and correspond to VP neurons. Given that the same GP neurons can increase or decrease firing rates depending on the specific inputs, it is possible that Type-II and III neurons may reflect different response patterns of the same neuronal population in the VP system. This possibility is further supported by the similar biophysical properties of Type-II and III neurons (Figures 5, 6).

In conclusion, our study presents the behavioral correlates and neurophysiological basis of three types of neurons in the BF region, and serves as a crucial initial step toward understanding the heterogeneous neuronal groups in the BF and VP region. Definitive answers to whether specific neuronal types belongs to BF corticopetal or ventral striatopallidal macrosystems will require future studies to determine the neurochemical identity and projection targets of these neurons.

## Conflict of interest statement

The authors declare that the research was conducted in the absence of any commercial or financial relationships that could be construed as a potential conflict of interest.
